# Non-invasive Estimation of Atrial Fibrillation Driver Position With Convolutional Neural Networks and Body Surface Potentials

**DOI:** 10.3389/fphys.2021.733449

**Published:** 2021-10-14

**Authors:** Miguel Ángel Cámara-Vázquez, Ismael Hernández-Romero, Eduardo Morgado-Reyes, Maria S. Guillem, Andreu M. Climent, Oscar Barquero-Pérez

**Affiliations:** ^1^Department of Signal Theory and Communications, Telematic Systems and Computation, Rey Juan Carlos University, Madrid, Spain; ^2^ITACA Institute, Universitat Politècnica de València, Valencia, Spain

**Keywords:** atrial fibrillation, body surface potentials, driver position, convolutional neural networks, deep learning

## Abstract

Atrial fibrillation (AF) is characterized by complex and irregular propagation patterns, and AF onset locations and drivers responsible for its perpetuation are the main targets for ablation procedures. ECG imaging (ECGI) has been demonstrated as a promising tool to identify AF drivers and guide ablation procedures, being able to reconstruct the electrophysiological activity on the heart surface by using a non-invasive recording of body surface potentials (BSP). However, the inverse problem of ECGI is ill-posed, and it requires accurate mathematical modeling of both atria and torso, mainly from CT or MR images. Several deep learning-based methods have been proposed to detect AF, but most of the AF-based studies do not include the estimation of ablation targets. In this study, we propose to model the location of AF drivers from BSP as a supervised classification problem using convolutional neural networks (CNN). Accuracy in the test set ranged between 0.75 (SNR = 5 dB) and 0.93 (SNR = 20 dB upward) when assuming time independence, but it worsened to 0.52 or lower when dividing AF models into blocks. Therefore, CNN could be a robust method that could help to non-invasively identify target regions for ablation in AF by using body surface potential mapping, avoiding the use of ECGI.

## 1. Introduction

Atrial fibrillation (AF) is the most common type of arrhythmia in clinical practice, affecting more than 33 million patients in the world (Chugh et al., 2014). AF is also a condition that increases the risk of the patients to suffer embolism, cardiac failure, stroke, and in the worst of cases, death (Fuster et al., 2006).

Therefore, one of the clinical goals in AF patients is to restore sinus rhythm. This objective can be achieved by pharmacological treatment (Lip and Tse, 2007), but termination of arrhythmic processes is usually accomplished by ablation of the cardiac tissue. Main targets of ablation are AF onset locations and drivers responsible for AF perpetuation (Guillem et al., 2016).

Previous human *in vivo* research showed different strategies to locate AF drivers and guide pulmonary veins isolation (PVI). In the case of invasive mapping procedures (Narayan et al., 2012; Krummen et al., 2017; Navara et al., 2018), several catheters are introduced inside the atrial chambers to record from 8 to 128 simultaneous electrograms (EGM) (Narayan et al., 2012). Despite the number of intracardiac signals, the large distance between catheter sensors (up to 1–2 cm) and the complex atrial anatomy limits the capability of intracardiac mapping systems to characterize the global electrical activity in AF (Oesterlein et al., 2016). Non-invasive procedures based on ECG imaging (ECGI) have been also tested to guide PVI (Haissaguerre et al., 2013, 2014; Dubois et al., 2015). However, PVI success rate gets lower in persistent AF, and phase singularity (PS)-guided ablation is suggested to be a reliable alternative for these more complicated cases (De Greef et al., 2018; Rottner et al., 2020).

Although ECGI has not been validated during AF propagation patterns, it has been demonstrated as a promising tool to identify AF drivers and guide ablation procedures (Cuculich et al., 2010; Haissaguerre et al., 2013, 2014; Pedrón-Torrecilla et al., 2016; Rodrigo et al., 2016). ECGI combines both numerical modeling of the bioelectric properties of the thorax and signal processing, with the aim of reconstructing the electrophysiological activity on the heart surface by using a non-invasive recording of body surface potentials (BSP) (Brooks and Macleod, 1997; Gulrajani, 1998). However, the inverse problem of ECGI has several drawbacks. First, it requires an accurate mathematical modeling of both atria and torso, mainly from CT or MR images. Next, the inverse problem of ECGI is ill-posed because the propagation between the epicardium and the torso implies information loss due to signal attenuation (Rodrigo et al., 2014), and BSP are also blurred compared to the signals on the heart due to the laws of electromagnetic field theory. Therefore, regularization methods are needed to obtain reliable and stable epicardial potential reconstructions (Tikhonov and Arsenin, 1977; Oster and Rudy, 1992; MacLeod and Brooks, 1998; Pedron-Torrecilla et al., 2011; Milanic et al., 2014). For these reasons, inverse problem-based approaches still need further improvement.

In the last decade, machine learning (ML) and deep learning (DL) techniques have undergone considerable development in bioengineering, and this include novel research in AF. For example, DL has been used in AF detection by using recurrent neural networks (RNN) and convolutional neural networks (CNN) (Xiong et al., 2017), by STFT, stationary wavelet transform and CNN (Xia et al., 2018), and in the detection of individuals at risk of suffering from Paroxysmal AF by CNN (Pourbabaee et al., 2018). However, most of AF-based studies do not include the estimation of ablation targets. Nonetheless, recent research showed that ML and DL methods can be also used in more complex tasks, like heart surface potentials estimation from BSP using autoencoders (Bujnarowski et al., 2020) and rotor identification from 12-lead ECG using decision trees (Luongo et al., 2021).

Therefore, in this study we propose to model the location of AF drivers from BSP as a supervised classification problem. We used CNN, which accounts for spatial characteristics, to address the location of AF drivers from previously labeled realistic computerized AF models (Figuera et al., 2016; Cámara-Vázquez et al., 2020).

The remaining of the study is organized as follows. In Section 2, we introduce the computational models used for this study, the experimental set-up, performance metrics, and DL architecture. Final results are summarized in sections 3 and in section 4 main conclusions are presented.

## 2. Materials and Methods

### 2.1. Computerized Models

**Geometric Models and EGM Computation**

Realistic 3D model for the atrial anatomy was composed of 284,578 nodes and 1,353,783 tetrahedrons (673.4 ± 130.3μ*m* between nodes) (Dössel et al., 2012) that considers a simplified single endocardium-epicardium layer for the atrial tissue.

We simulated 13 different AF propagation patterns in both left atria (LA) and right atria (RA), with different complexity and driver positions: posterior left atrial wall (PLAW), left inferior pulmonary vein (LIPV), left superior pulmonary vein (LSPV), right inferior pulmonary vein (RIPV), right superior pulmonary vein (RSPV), right atrial appendage (RAA), and right atria free wall (RAFW). Sampling rate of the signals was *fs* = 500*Hz*, while their duration ranged from 2 to 5 s.

The final computerized models were comprised of *N* = 2, 048 nodes for atria, and *M* between 2,206 and 3,970 nodes for torsos (10 different geometries were used), under the assumption of a homogeneous, unbounded, and quasi-static conducting medium by summing up all effective dipole contributions over the entire model (García-Molla et al., 2014; Figuera et al., 2016; Pedrón-Torrecilla et al., 2016; Rodrigo et al., 2017). Therefore, the EGMs of the entire model were computed as:


(1) $$\begin{array}{r} V\left(\vec{r}\right)=\underset{\vec{r}}{\sum }\left(\frac{\vec{r}}{r^{3}}\right)\cdot \vec{\nabla }\;V_{m} \end{array}$$

where $V\left(\vec{r}\right)$ is the EGM signal at the measuring point, *V*_*m*_ is the transmembrane potential distribution across the atria, $\vec{r}$ is the distance vector between the measuring point and a point in the tissue domain, and *r* is its corresponding scalar distance. The transmembrane potentials were defined in a scattered 3D mesh, so the gradient was computed by interpolating a quadratic function that involves two surrounding points (Lawson, 1984):


(2) $$\begin{array}{r} V_{m,i}-V_{m,j}=c_{1}x+c_{2}y+c_{3}z+c_{4}x^{2}+c_{5}y^{2}+c_{6}z^{2}+c_{7}xy+c_{8}yz+c_{9}xz \end{array}$$

where *V*_*m,i*_ and *V*_*m,j*_ are the transmembrane potentials at the points *i* and *j*; *x*, *y*, *z* are the incremental Cartesian coordinates from *j* to *i*, and coefficients *c*_1_ to *c*_9_ were computed by the least square method in, at least, nine neighboring points of each location.

**Atrial cell model**

The atrial cell model used is based on the one proposed in Nygren et al. (1998) and Koivumäki et al. (2014), where the electrical activity of a single myocyte is described in terms of their transmembrane potentials and ionic currents:


(3) $$\begin{array}{r} \frac{\partial V}{\partial t}=-\frac{I_{ion}}{C_{m}} \end{array}$$

where *V* is the transmembrane potential, *I*_*ion*_ is the transmembrane ionic currents, and *C*_*m*_ is the cell membrane capacitance.

Then, the electrical propagation across the atrial tissue was simulated by including the transmembrane currents caused by the intercellular Gap junction current (due to the transmembrane potential gradient) in the previous formulation:


(4) $$\frac{\partial V_{k}}{\partial t}=-\frac{I_{ion}}{C_{m}}-\sum_{i=1}^{N}D_{K,i}\frac{V_{k}-V_{i}}{d_{k,i}^{2}}$$

where *V*_*k*_ is the transmembrane potential at node *k*, *V*_*i*_ is the transmembrane potential at the neighbor node *i*, *D*_*k,i*_ is the diffusion coefficient between the node *k* and *i*, and *d*_*k,i*_ is the distance between those nodes. Since the atrial electrical conduction is anisotropic (its velocity is higher in the longitudinal fiber orientation than at transverse), the diffusion coefficient *D*_*k,i*_ that modulates the intercellular ionic current is determined as follows:


(5) $$\begin{array}{r} D_{K,i}=D_{long}\cdot cos^{2}\alpha +D_{trans}\cdot sin^{2}\alpha \end{array}$$

where α is the angle between the longitudinal fiber orientation and the vector linking nodes *k* and *i*, and *D*_*long*_ and *D*_*trans*_ are the longitudinal and transverse diffusion coefficients, respectively (Rodrigo, 2016). Fibrotic and scar tissues were modeled by setting the diffusion values of the involved nodes to 0. In the case of the fibrotic tissue, a certain percentage of random nodes were disconnected, depending on the pattern to simulate. The final system of differential equations was solved by Runge-Kutta integration (using NVIDIA Tesla C2075 6G).

### 2.2. Input Data

#### 2.2.1. Atrial Fibrillation Driver Detection as a ML Classification Problem

**Figure 1:**
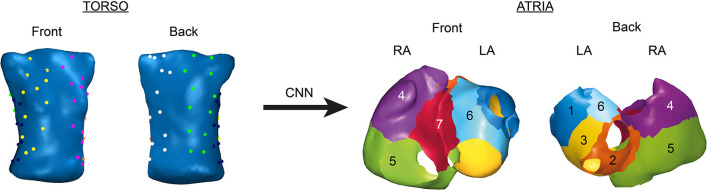
Position of the electrodes for body surface potential mapping (BSPM, left) and regions in atria where AF drivers can be found (right).

We proposed to address the location of AF drivers as a supervised classification problem. We divided the atria into seven regions (Figure 1, right) where the AF driver can be found (Haissaguerre et al., 2014). Each region, which ranged from 1 to 7, represents a class to which the AF driver belongs. To obtain labeled data, AF driver location from the computerized model was manually classified into one region for each time-instant. An additional label (0) is assigned when no driver is found.

#### 2.2.2. Body Surface Potentials (BSP)

Input data for DL algorithms are simulated BSP from each of the 10 torso geometries available. To compute the initial BSP ***y***, the forward problem of ECGI was solved by computing the corresponding *M* × *N* transfer matrix *A* using the boundary element method (Barr et al., 1977; De Munck, 1992; Pedrón-Torrecilla et al., 2016):


(6) $$\begin{array}{r} y_{t}=Ax_{t} \end{array}$$

where *y*_*t*_ are the BSP from each time instants. Those simulated BSP must be referenced to the Wilson Central Terminal (WCT). This step is essential since real ECG recordings are referenced to this point due to the electrical noise of the ground, and it can be mathematically represented as:


(7) $$\begin{array}{r} ECG=ECG_{notref}-ECG_{WCT} \end{array}$$

where *ECG*_*notref*_ is the not-corrected ECG, *ECG*_*WCT*_ is the WCT signal, computed as the average of the ECG signals at the WCT points, and *ECG* is the final corrected ECG. Therefore, if we apply the same methodology to BSP, *y*_*t*_ referenced signals can be computed as:


(8) $$\begin{array}{r} y_{t,ref}=Ax_{t}-\frac{1}{N_{WCT}}\underset{N\in WCT}{\sum }Ax_{t}=Ax_{t}-\frac{1}{N_{WCT}}M_{WCT}x_{t}= \end{array}$$

(9) $$\begin{array}{r} =\left(A-\frac{1}{N_{WCT}}M_{WCT}\right)x_{t} \end{array}$$

where *N*_*WCT*_ is the number of WCT points, and *M*_*WCT*_ is a matrix of zeros except the rows that correspond to the WCT leads, that have the same values as the corresponding rows of the *A* matrix (Rodrigo, 2016). Therefore, to directly compute the BSP referenced to the WCT, it is possible to compute a corrected *A*_*CTW*_ matrix as:


(10) $$\begin{array}{r} A_{WCT}=A-\frac{1}{N_{WCT}}M_{WCT} \end{array}$$

Once the WCT-referenced simulated BSP were computed, they were corrupted with additive Gaussian noise and filtered using: fourth-order bandpass Butterworth filter (fc_1_ = 3 Hz and fc_2_ = 30 Hz) (Figuera et al., 2016; Pedrón-Torrecilla et al., 2016). Signal-to-noise ratios (SNR) ranged from 5 to 50 dB. Finally, a set of 64 electrodes from each torso were chosen to represent a realistic multi-electrode vest used in electrophysiological studies, as shown in Figure 1 (left).

#### 2.2.3. Image Representation of BSP

**Figure 2:**
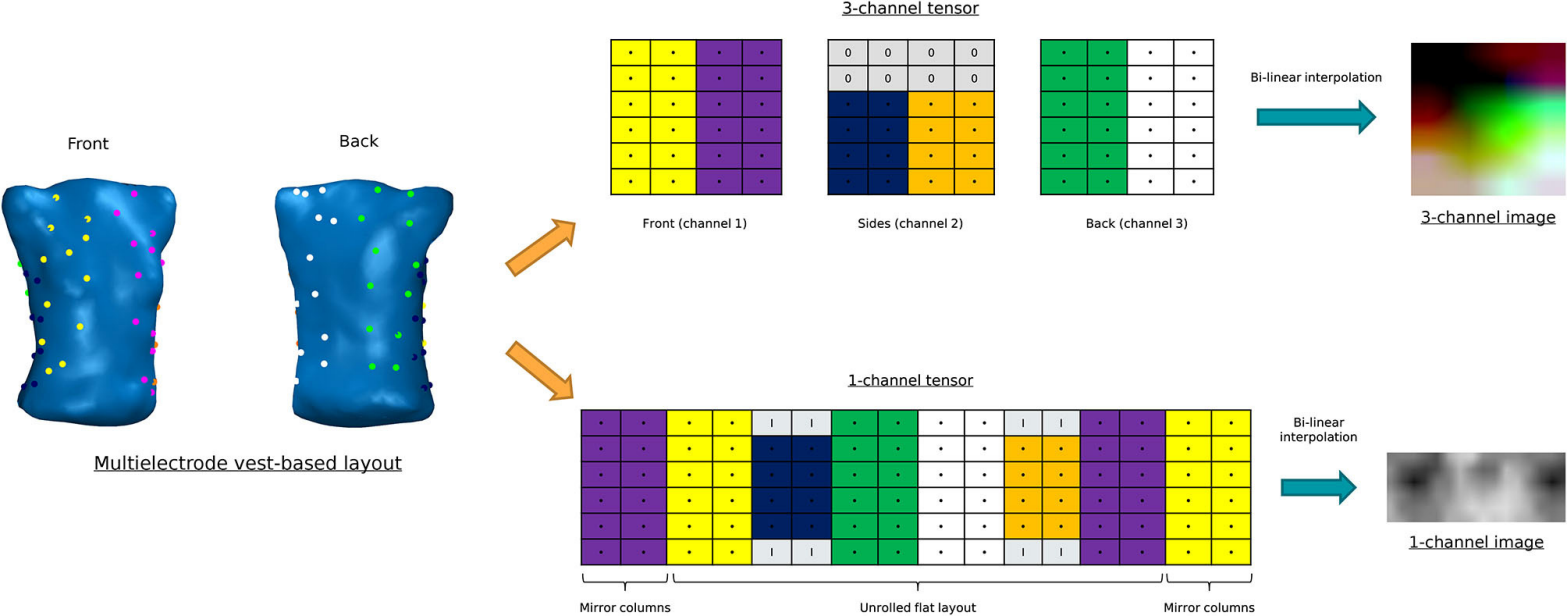
Construction of the different tensor layouts. Above: 3-channel tensor. Below: 1-channel tensor.

To obtain final input data, we represented the layout of electrodes in Figure 1 as images. For this purpose, we build two different types of tensors for each time instant (see Figure 2):

- 3-channel tensor. This first approach consists on creating 3D matrices of shape (6 × 4 × 3). The first and last channel contain the BSP of the torso and back, respectively (24 electrodes each), whereas the second channel contains BSP from the sides (16 electrodes distributed on the last 4 rows, while the rest are filled with zeroes).
- 1-channel tensor. This second approach consists on organizing the BSP in a single-channel 2D matrix of shape (6 × 16 × 1). To do that, we considered a multielectrode vest as a cylinder that was unrolled to a 12-column flat layout. Then, we added two additional mirror columns to each side of the matrix to represent the fact that the first and last columns of the tensor are in touch in a real 3D geometry. Finally, since sides electrodes are four for each column (instead of 6), the empty values are filled with the mean of the three nearest electrodes.

Finally, to increase the size of the images, we performed a bilinear interpolation to create tensors with shape (150 × 152 × 3) and (78 × 192 × 1), respectively.

### 2.3. Convolutional Neural Network (CNN) Architectures

#### 2.3.1. Custom CNN

CNN are a type of DL algorithm used mainly in image recognition and image classification. Comparing with other types of DL algorithms, this approach has a superior ability to extract features, increasing classification performance (Krizhevsky et al., 2017).

CNN-based architectures consist on the following layers:

- **Input layer**. In this case, input data are an image, i.e., a 3D matrix. This input matrix is also named as tensor.
- **Convolutional layer**. In this type of layer, a filter of size (*s, t*) is applied to the input tensor. The main aim of the convolutional layer is to extract feature maps that can reflect certain characteristics of interest (edges, shape detection, etc.) (Li et al., 2019). This filtering process is performed using the convolution operation, which can be mathematically represented as:

(11) $$\begin{array}{r} a_{i,j}=f\left(\overset{s}{\underset{m=0}{\sum }}\overset{t}{\underset{n=0}{\sum }}w_{m,n}x_{i+m,j+n}+b\right) \end{array}$$

where *a*_*i,j*_ is the output (activation) on coordinates (*i,j*), *x*_*i*+*m, j*+*n*_ is the value of the input tensor in coordinates (*i*+*m, j*+*n*), *w*_*m,n*_ is the coefficient of the filter in coordinates (*m, n*), *b* is the bias, and *f*(·) is a nonlinear activation function.

- **Pooling layer**. This type of layers is frequently used between convolutional layers, since they reduce the amount of information that convolutional layers generate. There are several ways to implement a pooling layer, and one of the most used is the max-pooling. In this case, a non-overlapped window is applied to the input feature map, and the output is the maximum value of the considered window. If we consider a square window, the output feature map shape will be reduced in a 1/*m* factor, where *m* is the dimension of the window.
- **Dense layer**. After applying a certain combination of convolutional and pooling layers, a multilayer perceptron (MLP) can be applied afterwards. Therefore, it is necessary to create an unidimensional vector from the output of the convolutional network (flatten operation). As explained before, the output layer of the MLP will have one unit for each value the network should output.

**Figure 3:**
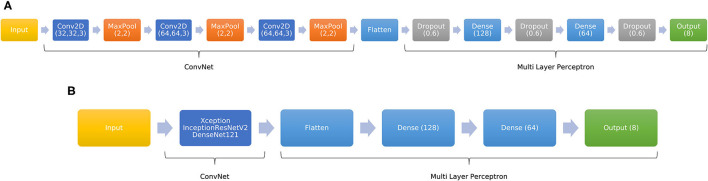
Schematic overview of the CNN-based proposed architectures. **(A)** Custom CNN. **(B)** Adaptation of pre-trained network to a 8-unit output.

The proposed CNN-based architecture is shown in Figure 3A. Input data are tensors of shape (150 × 152 × 3) or (78 × 192 × 1), depending on the tensor architecture evaluated. We used three convolutional layers with 32, 64 and 64 filters with (3 × 3) size. ReLU activation function was used in both convolutional layers. Max-pooling is applied after each convolutional layer [(2,2) window size]. Final dense layers are composed by layers of (128, 64) units (ReLU activation function), while the output layer is a Softmax layer composed by 8 units, one for each label. Finally, we used dropout between dense layers (0.6 dropout rate) to avoid overfitting.

#### 2.3.2. Pre-trained CNN

Finding the right parameters for a CNN (number and dimensions of layers, pooling, etc.) can be a very complicated task, and training this type of network is also very computationally expensive. To address this task, there are several models that were previously trained with a certain dataset that can be also used to solve similar problems. This approach is called transfer learning (Pan and Yang, 2009).

In this study, we decided to adapt the DenseNet121 (DN121, Huang et al., 2016), InceptionResNetV2 (IRNV2, Szegedy et al., 2016), and Xception (Chollet, 2016) models to our problem. These models were trained with the ImageNet database (Russakovsky et al., 2015), comprised of 14 million images corresponding to 1,000 different classes. Therefore, since we want to obtain an output with the prediction for 8 cases instead of 1,000, we added several fully connected layers to the output of the pre-trained models. This adaptation is showed in Figure 3B. Dense layers were composed of (128,64) units (ReLU activation function), while the output layer is a Softmax layer composed by 8 units.

Finally, it is essential to remark that these pre-trained models require 3-channel input tensors. Therefore, to use the previously described 1-channel tensor, we decided to repeat the 2D matrix twice to obtain a 3-channel tensor [(78 × 192 × 3) shape].

### 2.4. Performance Metrics

To assess the performance of the implemented DL models, we used four different metrics:

- Accuracy (*Acc*). It is measured as
  (12) $$\begin{array}{r} Acc=\frac{TP_{full}}{Total} \end{array}$$
  where *TP*_*full*_ is the total number of well-classified drivers (true positives), and *Total* is the total number of drivers.
- Cohen's Kappa (κ). It is a robust statistic used for rating reliability testing (McHugh, 2012), and is computed as
  (13) $$\begin{array}{r} \kappa =\frac{p_{o}-p_{e}}{1-p_{e}} \end{array}$$
  where *p*_*o*_ is the relative agreement among raters (identical to accuracy), and *p*_*e*_ the hypothetical probability of chance agreement. A score of 1 represents a perfect agreement between raters, and a score of 0 represents the agreement that can be expected from random chance. Scores less than 0 mean that there is less agreement than chance.
- Sensitivity (or true positive rate, TPR). It is the proportion of positive samples for a class that are correctly classified. It is measured as:
  (14) $$\begin{array}{r} TPR=\frac{TP}{TP+FN} \end{array}$$
  where *TP* is the number of well-classified drivers for a certain class (true positives), and *FN* is the number of drivers that was wrongly classified to another class (false negatives). It is considered a measure of how well a test can identify true positives.
- Specificity (or true negative rate, TNR). It is the proportion of negative samples for a class that are correctly classified. It is measured as:
  (15) $$\begin{array}{r} TNR=\frac{TN}{TN+FP} \end{array}$$
  where *TN* is the number of drivers that did not belong to a certain class that were correctly classified (true negatives), and *FP* is the number of drivers that was classified as positive for the same class, but they correspond to another class (false positives). It is considered a measure of how well a test can identify true negatives.

### 2.5. Experimental Set-Up

Final data set is composed of input data tensors, ***x*_*i*_**, of shape (150 × 152 × 3) or (78 × 192 × 1) [(78 × 192 × 3) in the case of the pre-trained models], obtained from 64 BSP electrodes, and their corresponding label *y*_*i*_, which can have values 0, 1, …, 7.

Training and test sets were split using two different approaches:

- Time independence of input data. In this case, each time instant is considered as an independent sample. The whole data set was then randomly split into training (80%) and test (20%) sets, using hold-out validation during the training process (20%). In this scenario, samples from each AF model are randomly distributed to the training, validation, and test sets, but training, validation, and test processes are carried out with completely different samples, i.e., samples from the training set are not used in the validation and test sets.
- Division of AF models in consecutive blocks. To describe a more realistic scenario, we divided each AF computerized model in three independent blocks: training (first 80% of the length of the signals), hold-out validation (last 20% of the training set), and test (final 20% of the length of the signals). Therefore, these three blocks contain consecutive samples, i.e., the training process is carried out using the first block of time instants, and test is achieved with the last block of samples.

On the other hand, we had to address imbalance of data. In our computerized models, there are several atrial areas where the driver only appears in one single AF computerized model, whereas other areas are more susceptible of containing a driver. Therefore, there are several atria regions that are over represented in the data set. To address this problem, we tried to weigh the classes accordingly to the probability of occurrence (class weight). It applies a higher penalization in wrong classifications in under-represented classes.

Regarding the training process, we trained the different models for a maximum number of epochs of 1,000, with reduction in the learning rate and early stopping if there is no improvement in the loss value during a certain number of training epochs. Moreover, we use the model checkpoint technique to get the best model obtained during the training process according to a metric of interest (loss value for the custom CNN, and validation loss for the pre-trained CNN). Finally, in the case of the pre-trained models, we followed two different training approaches: training the dense layers and the last two convolutional layers (while freezing the rest of the CNN, partial training), and re-training the whole pre-trained network (full training).

## 3. Results

### 3.1. Performance of CNN

The first carried out experiment consisted on assessing the obtained performance with each tensor architecture. For this purpose, we trained the CNN architecture explained in Figure 3 with 1-channel and 3-channel tensors. In both cases, tensors were obtained from the same BSP dataset. Therefore, we got two different CNN models to be compared. Moreover, to simulate a realistic scenario, we used noisy BSP (SNR = 20 dB).

Table 1 shows the results considering each time instant as an independent sample and division in blocks. In the first case, we were able to correctly locate the 95 and 91% of drivers in the training and test sets, respectively (Cohen's kappa of 0.92 and 0.88) when using 3-channel tensors. Results obtained with the CNN model trained with 1-channel tensors were very similar, with an accuracy of 0.94 and 0.91 in training and test sets, respectively (Cohen's kappa of 0.92 and 0.88).

**Table 1:** Performance metrics obtained with CNN assuming independent time instants and division in blocks.

	**Independent time instants**		**Division in blocks**	
	**3-channel tensor**	**1-channel tensor**	**3-channel tensor**	**1-channel tensor**
Training accuracy	0.954	0.942	0.971	0.965
Training Kappa	0.940	0.924	0.962	0.955
Test accuracy	0.913	0.912	0.499	0.526
Test Kappa	0.887	0.886	0.336	0.373

On the other hand, results for division in blocks were worse. In this scenario, accuracy was 0.97 and 0.49 in training and test sets when training with 3-channel tensors, respectively (Cohen's kappa of 0.96 and 0.33). Metrics when training with 1-channel tensors were similar in the training set but slightly better in the test set (accuracy of 0.52 and Cohen's kappa of 0.37), but worse compared with time independence approach. These results suggest that we are overfitting the model to the training set.

**Figure 4:**
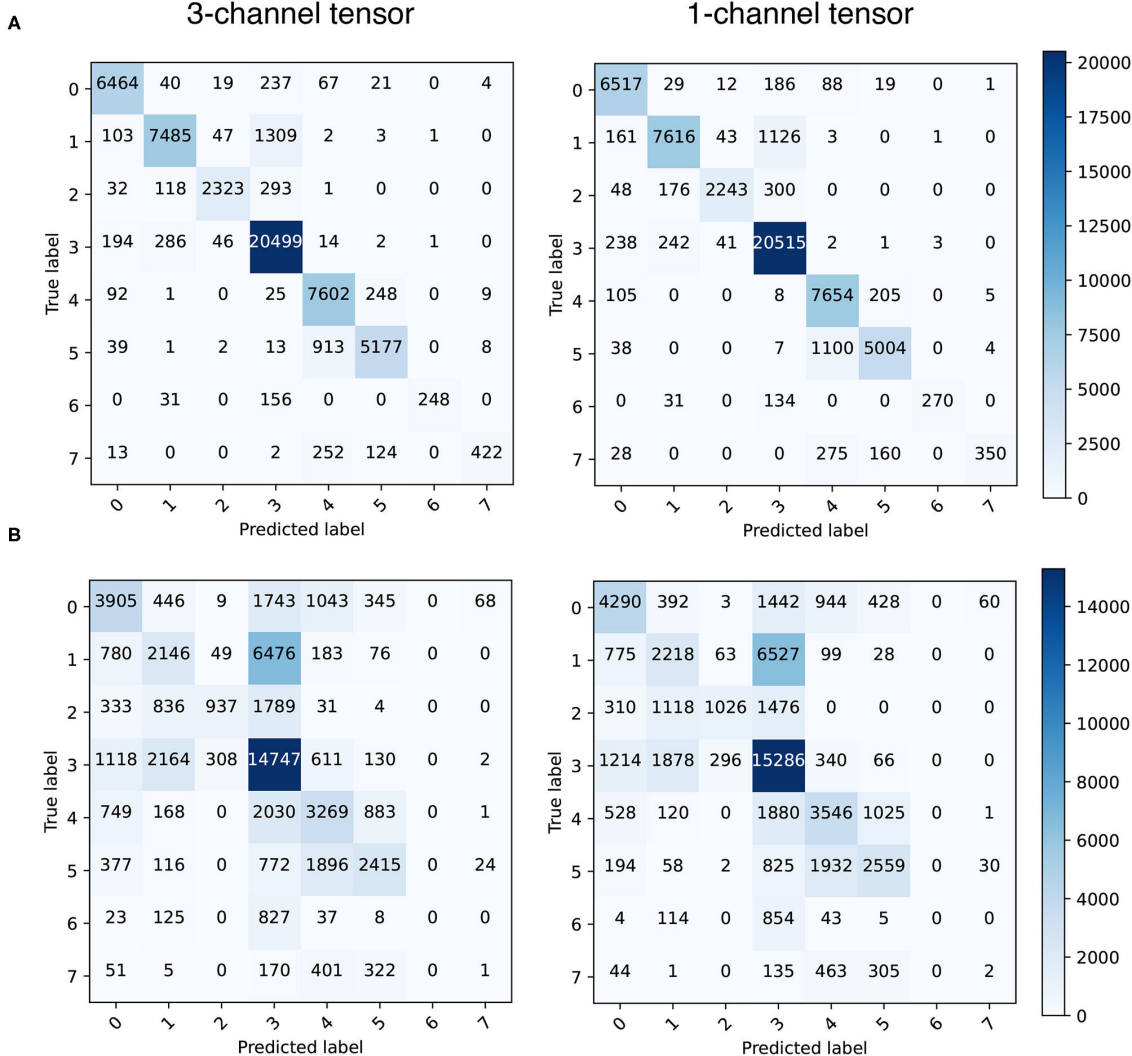
Confusion matrices obtained in the test set (1-channel tensor) for time independence **(A)** and division in blocks **(B)**.

Figure 4 shows the confusion matrix obtained in the test set for both training approaches and tensor types, while Table 2 shows the accuracy, sensitivity and specificity obtained for each label when training with 1-channel tensors. In the case of time independence, accuracy ranged from 0.81 to 0.975, although it worsened to 0.43 in the case of the drivers located in the septum area (label 7, accuracy of 0.430). These results are justified by the imbalance of our dataset. Labels from 0 to 5, although their population are also imbalanced, are highly represented, but the number of samples with labels 6 and 7 is very low. Results for the CNN model trained with 3-channel tensors were slightly worse. Regarding sensitivity, the system is able to detect with high precision clinically relevant regions. Those areas where ECGI has less reconstruction capacity (like the septum area), the sensitivity is lower. However, it is essential to remark that both in clinical practice and in our database these regions present a lower probability of present drivers. Regarding specificity, obtained scores were always above 0.94.

**Table 2:** Accuracy, sensitivity, and specificity scores obtained with 1-channel CNN assuming time independence and division in blocks (by labels, test set).

	**Atrial region (time independence)**							
	**No driver (0)**	**1**	**2**	**3**	**4**	**5**	**6**	**7**
Accuracy	0.951	0.851	0.810	0.975	0.959	0.813	0.620	0.430
Sensitivity (TPR)	0.951	0.850	0.810	0.974	0.959	0.813	0.620	0.430
Specificity (TNR)	0.987	0.989	0.998	0.948	0.968	0.992	0.999	0.999
	**Atrial region (division in blocks)**							
	**No driver (0)**	**1**	**2**	**3**	**4**	**5**	**6**	**7**
Accuracy	0.567	0.228	0.261	0.801	0.499	0.457	0	0.002
Sensitivity (TPR)	0.569	0.231	0.268	0.799	0.494	0.457	0	0.001
Specificity (TNR)	0.935	0.918	0.993	0.635	0.918	0.962	1	0.998

In the case of division in blocks, results were significantly worse. Highest accuracy was obtained for the label 3 (0.80), which is the one with the largest representation in the dataset. However, accuracy for the rest of labels ranged from 0.22 to 0.56, and near 0 for labels 6 and 7. The high temporal redundancy between consecutive signal samples could explain overfitting in this scenario, although the training set was previously shuffled before fitting the model. The low number of computerized AF models makes also difficult to help the CNN model to generalize. Regarding sensitivity, results were also significantly worse, although the system was able to get a sensitivity of 0.799 in the most represented atrial zone (label 3). Finally, the rate of TN is very high for every label, except for the label 3, with a specificity of 0.635.

### 3.2. Performance of Pre-trained CNN and Noise Robustness

In this second experiment, we evaluated the possibility of using pre-trained CNN models for AF driver localization, instead of using a custom CNN architecture. Models were trained with 1-channel tensors obtained with noisy BSP (SNR = 20 dB). Since pre-trained models require 3-channel tensors, we repeated the tensor twice to obtain 3-channel tensors. The custom CNN model was trained with the original 1-channel tensor.

Moreover, to assess the noise robustness of those DL models, we tested them with tensors whose associated BSP were corrupted with noise (SNR from 5 to 50 dB, in steps of 5 dB). This procedure was repeated 20 times in order to obtain test signals with different noise but same SNR. Then, the mean and SD of performance metrics were computed.

**Figure 5:**
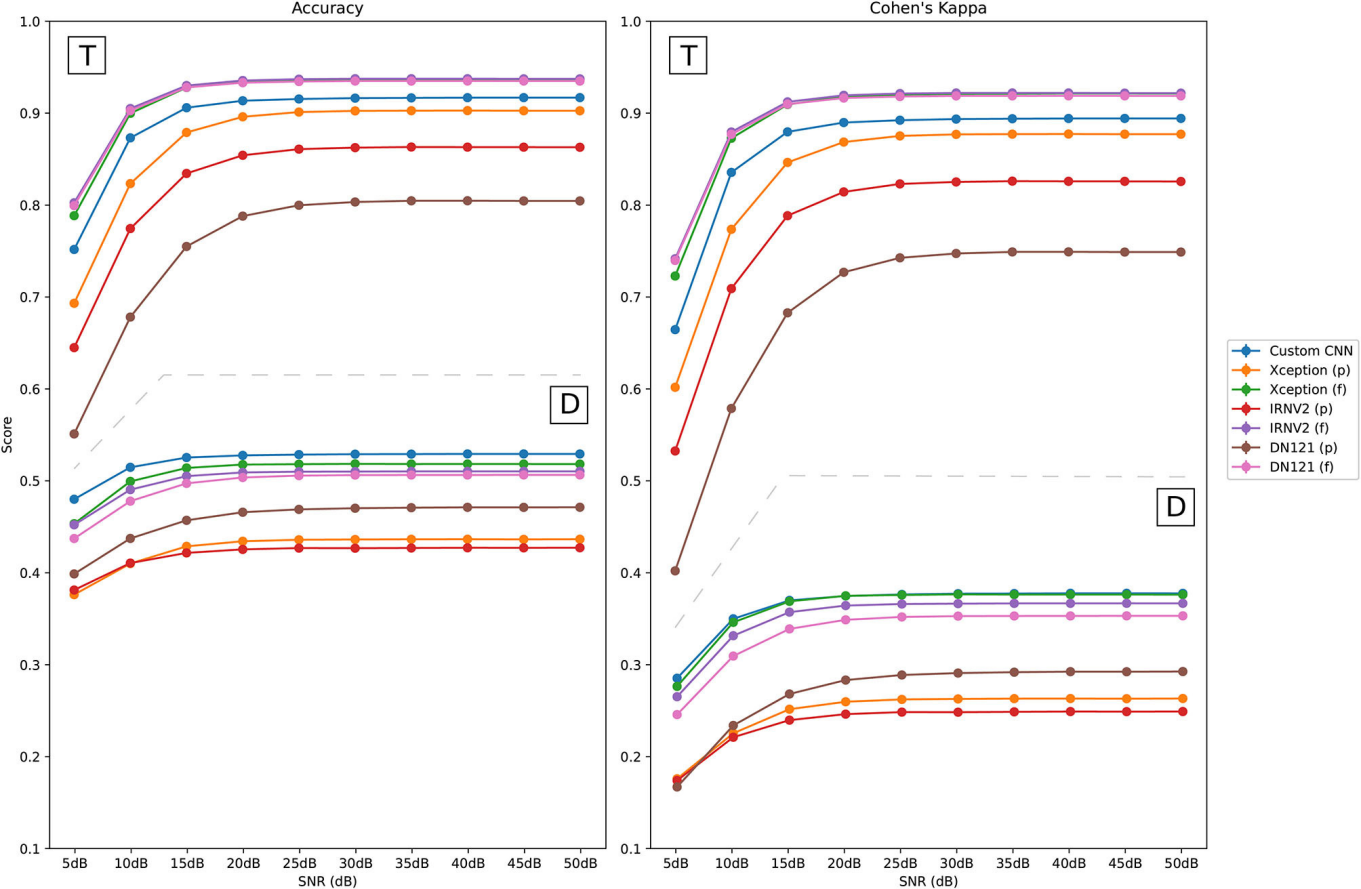
Performance of CNN models on noisy test signals when assuming time independence (T) and division in blocks (D). Partially trained CNN models are marked with (p), and fully trained ones are identified as (f).

Figure 5 shows the performance metrics obtained for the test set, assuming time independence and division in blocks, for each CNN model, whereas Table 3 shows the average accuracy values for the custom CNN model and fully trained pre-trained models (SNR from 5 to 20 dB). In both cases, performance of pre-trained networks was significantly better when training the full network instead of performing a partial training (where the network was freezed except the last two convolutional layers and the fully connected ones). For time independence, average accuracy values obtained with fully trained pre-trained models ranged from 0.788 (5 dB) to 0.93 (20 dB upward), whereas the best accuracy value obtained for partially trained models was 0.90 (Xception model). Regarding average Cohen's kappa metric, it ranged from 0.72 (5dB) to 0.91 (20 dB upward) for fully-trained models, while the highest obtained score for all partially trained pre-trained models was 0.87 (Xception model).

**Table 3:** Average accuracy values obtained for the different fully trained networks and noisy PSD (SNR = 5, 10, 15 and 20 dB, test set).

	**Independent time instants**			
	**5 dB**	**10 dB**	**15 dB**	**20 dB**
Custom CNN	0.751 ± 0.001	0.872 ± 0.001	0.905 ± 0.0005	0.912 ± 0.0003
Xception (f)	0.788 ± 0.001	0.899 ± 0.001	0.927 ± 0.0007	0.933 ± 0.0005
InceptionResNetV2 (f)	0.802 ± 0.001	0.904 ± 0.001	0.929 ± 0.0008	0.934 ± 0.0003
DenseNet121 (f)	0.799 ± 0.001	0.902 ± 0.001	0.927 ± 0.0006	0.932 ± 0.0005
	**Division in blocks**			
	**5 dB**	**10 dB**	**15 dB**	**20 dB**
Custom CNN	0.479 ± 0.002	0.514 ± 0.001	0.524 ± 0.001	0.527 ± 0.001
Xception (f)	0.453 ± 0.003	0.498 ± 0.002	0.513 ± 0.001	0.517 ± 0.0006
InceptionResNetV2 (f)	0.452 ± 0.002	0.490 ± 0.002	0.504 ± 0.001	0.508 ± 0.0006
DenseNet121 (f)	0.437 ± 0.002	0.477 ± 0.001	0.496 ± 0.001	0.503 ± 0.001

On the other hand, average accuracy values obtained for the division in blocks approach were significantly worse for all the CNN models. The best average accuracy value was 0.51 among the different pre-trained models (SNR = 20 dB upward, Xception fully trained model), whereas the best overall accuracy value was 0.527 for the custom CNN model. Both are much lower values than the ones obtained for time independence.

Regarding the performance when testing with much noisier signals, performance degraded for SNR = 5 dB, with average accuracy scores below 0.802 when assuming time independence (0.479 with division in blocks). However, it improved from SNR = 10 dB with average accuracy values over 0.87 in the time independence approach (in the division in blocks approach the best score for SNR = 10 dB was 0.51 for the custom CNN model). Additionally, SD values of the accuracy were always below 0.001, which suggests that the CNN models are robust to noise changes.

Finally, performance metrics for fully trained pre-trained models were very similar between them when assuming time independence, outperforming the custom CNN model. However, in the case of division in blocks, the custom CNN outperformed fully trained pre-trained networks, although differences between these models were very small.

## 4. Discussion and Conclusions

The use of CNNs can help to identify target regions for ablation using body surface potential mapping, avoiding ECGI. The proposed method, which converts BSP into images, has been demonstrated to be accurate and robust to noise, i.e., the performance just degrades for very low values of SNR.

Regarding the architecture of the tensors, the 1-channel tensor-based architecture was able to obtain more accurate results than the 3-channel one, both assuming time independence or division in blocks. In relation to the training process, the single-channel tensor-based CNN model required a higher number of epochs to be fitted than in the 3-channel one. However, since each epoch is slower in the 3-channel architecture (3D matrix), the final training process becomes faster when using single-channel tensors.

Moreover, this methodology makes transfer learning very easy to apply, since it can be used to adapt much more complex pre-trained CNN models to a very specific task with promising results. However, results were significantly better when re-training the whole network (much slower procedure) than when training just the final layers. Therefore, using pre-trained models requires further research.

Although this study showed very promising results, it has several limitations that should be taken into account. The first one is the size and balance of our dataset. It is composed by 130 sets of BSP, obtained with 10 torso geometries but from only 13 AF computerized models, so the number of represented propagation patterns is low. Moreover, the distribution of drivers across the seven defined atrial regions is not balanced, i.e., there are regions that are over-represented, and performance will be worse on under-represented regions. However, we have decided to use the proposed division in seven regions because it represents a clinical-based classification of the areas where the AF drivers are more commonly found and has been already clinically used to guide AF ablation strategies demonstrating to have a clear clinical significance (Haissaguerre et al., 2014). Besides, the proposed classification method can be easily extrapolated to other atrial geometry divisions based on a higher number of smaller regions, being able to get a higher resolution in the driver classification.

Another important problem to be faced is the training approach. In this study, we first trained the models with random samples that belongs to the 13 AF models available (time independence). However, in a real situation, the DL model will have to compute a prediction with a data set that the network has never seen. Therefore, we decided to train the models with a chunk of signals of each model, while testing with the rest. In this scenario, performance substantially worsened because of the high temporal redundancy between consecutive time instants and the low number of AF models. Both facts lead to overfit to the training set, even when using tools like dropout. Using RNNs could be useful to face the redundancy problem, since they are able to extract temporal characteristics of BSP that are omitted when using CNN.

In a real scenario, assuming model independence (i.e., training with some AF models and testing with the rest) should be the way to go, but it will require the availability of a higher number of computerized models and propagation patterns. Finally, another point of future work will be applying this methodology with real patient data. However, nowadays there is a lack of gold standard for the identification of AF drivers, and there are not labeled clinical data that could help us to validate this methodology.

## Data Availability Statement

Some of the computerized models used in our study are publicly available at the Experimental Data and Geometric Analysis Repository (EDGAR, https://edgar.sci.utah.edu/) by the Consortium for ECG Imaging (CEI, https://www.ecg-imaging.org/). Further inquiries can be directed to the corresponding authors.

## Author Contributions

MÁC-V and OB-P: experimental setup, code implementation, and computational tests. MÁC-V, IH-R, MG, AC, and OB-P: data collecting, cleaning, and pre-processing. MÁC-V, IH-R, EM-R, MG, AC, and OB-P: manuscript preparation. All authors contributed to the article and approved the submitted version.

## Funding

This work has been partially supported by: Ministerio de Ciencia e Innovación (PID2019-105032GB-I00), Instituto de Salud Carlos III, and Ministerio de Ciencia, Innovación y Universidades (supported by FEDER Fondo Europeo de Desarrollo Regional PI17/01106 and RYC2018-024346B-750), Consejería de Ciencia, Universidades e Innovación of the Comunidad de Madrid through the program RIS3 (S-2020/L2-622), EIT Health (Activity code 19600, EIT Health is supported by EIT, a body of the European Union) and the European Union's Horizon 2020 research and innovation program under the Marie Skłodowska-Curie grant agreement No. 860974.

## Conflict of Interest

AC, MG, and IH-R hold equity in Corify Care. AC have received honoraria from Corify Care. The remaining authors declare that the research was conducted in the absence of any commercial or financial relationships that could be construed as a potential conflict of interest.

## Publisher's Note

All claims expressed in this article are solely those of the authors and do not necessarily represent those of their affiliated organizations, or those of the publisher, the editors and the reviewers. Any product that may be evaluated in this article, or claim that may be made by its manufacturer, is not guaranteed or endorsed by the publisher.

## References

[B1] Barr R. C. Ramsey M. Spach M. S. (1977). Relating epicardial to body surface potential distributions by means of transfer coefficients based on geometry measurements. IEEE Trans. Biomed. Eng. 24, 1–11. 10.1109/TBME.1977.326201 832882

[B2] Brooks D. H. Macleod R. (1997). Electrical imaging of the heart. IEEE Signal Process. Mag. 14, 24–42. 10.1109/79.560322

[B3] Bujnarowski K. Bonizzi P. Cluitmans M. Peeters R. Karel J. (2020). Ct-scan free neural network-based reconstruction of heart surface potentials from ecg recordings, in 2020 Computing in Cardiology (Rimini: IEEE), 1–4.

[B4] Cámara-Vázquez M. Á Oter-Astillero A. Hernández-Romero I. Rodrigo M. Morgado-Reyes E.. (2020). Atrial fibrillation driver localization from body surface potentials using deep learning, in 2020 Computing in Cardiology (Rimini,: IEEE), 1–4.

[B5] Chollet F. (2016). Xception: Deep learning with depthwise separable convolutions. CoRR, abs/1610.02357. 10.1109/CVPR.2017.195

[B6] Chugh S. S. Havmoeller R. Narayanan K. Singh D. Rienstra M. Benjamin E. J.. (2014). Worldwide epidemiology of atrial fibrillation: a global burden of disease 2010 study. Circulation 129, 837–847. 10.1161/CIRCULATIONAHA.113.005119 24345399 PMC4151302

[B7] Cuculich P. S. Wang Y. Lindsay B. D. Faddis M. N. Schuessler R. B. Damiano R. J.. (2010). Noninvasive characterization of epicardial activation in humans with diverse atrial fibrillation patterns. Circulation 122, 1364–1372. 10.1161/CIRCULATIONAHA.110.945709 20855661 PMC2996091

[B8] De Greef Y. Schwagten B. Chierchia G. B. de Asmundis C. Stockman D. Buysschaert I. (2018). Diagnosis-to-ablation time as a predictor of success: early choice for pulmonary vein isolation and long-term outcome in atrial fibrillation: results from the Middelheim-PVI Registry. EP Eur. 20, 589–595. 10.1093/europace/euw426 28340103

[B9] De Munck J. (1992). A linear discretization of the volume conductor boundary integral equation using analytically integrated elements. IEEE Trans. Biomed. Eng. 39, 986–990. 10.1109/10.256433 1473829

[B10] Dössel O. Krueger M. W. Weber F. M. Wilhelms M. Seemann G. (2012). Computational modeling of the human atrial anatomy and electrophysiology. Med. Biol. Eng. Comput. 50, 773–799. 10.1007/s11517-012-0924-6 22718317

[B11] Dubois R. Shah A. J. Hocini M. Denis A. Derval N. Cochet H.. (2015). Non-invasive cardiac mapping in clinical practice: application to the ablation of cardiac arrhythmias. J. Electrocardiol. 48, 966–974. 10.1016/j.jelectrocard.2015.08.028 26403066

[B12] Figuera C. Suárez-Gutiérrez V. Hernández-Romero I. Rodrigo M. Liberos A. Atienza F.. (2016). Regularization techniques for ECG Imaging during atrial fibrillation: a computational study. Front. Physiol. 7:466. 10.3389/fphys.2016.00556 27790158 PMC5064166

[B13] Fuster V. Rydén L. E. Cannom D. S. Crijns H. J. Curtis A. B. Ellenbogen K. A.. (2006). Acc/aha/esc 2006 guidelines for the management of patients with atrial fibrillation: full text. Europace 8, 651–745. 10.1093/europace/eul097 16987906

[B14] García-Molla V. Liberos A. Vidal A. Guillem M. Millet J. González A.. (2014). Adaptive step {ODE} algorithms for the 3d simulation of electric heart activity with graphics processing units. Comput. Biol. Med. 44, 15–26. 10.1016/j.compbiomed.2013.10.023 24377685

[B15] Guillem M. Climent A. Rodrigo M. Fernández-Avilés F. Atienza F. Berenfeld O. (2016). Presence and stability of rotors in atrial fibrillation: evidence and therapeutic implications. Cardiovasc. Res. 109, 480–492. 10.1093/cvr/cvw011 26786157 PMC4777913

[B16] Gulrajani R. (1998). The forward and inverse problems of electrocardiography. IEEE Eng. Med. Biol. 17, 84–101. 10.1109/51.715491 9770610

[B17] Haissaguerre M. Hocini M. Denis A. Shah A. J. Komatsu Y. Yamashita S.. (2014). Driver domains in persistent atrial fibrillation. Circulation 130, 530–538. 10.1161/CIRCULATIONAHA.113.005421 25028391

[B18] Haissaguerre M. Hocini M. Shah A. J. Derval N. Sacher F. Jais P.. (2013). Noninvasive panoramic mapping of human atrial fibrillation mechanisms: a feasibility report. J. Cardiovasc. Electrophysiol. 24, 711–717. 10.1111/jce.12075 23373588

[B19] Huang G. Liu Z. Weinberger K. Q. (2016). Densely connected convolutional networks. CoRR, abs/1608.06993. 10.1109/CVPR.2017.243

[B20] Koivumäki J. T. Seemann G. Maleckar M. M. Tavi P. (2014). *In silico* screening of the key cellular remodeling targets in chronic atrial fibrillation. PLoS Comput. Biol. 10:e1003620. 10.1371/journal.pcbi.1003620 24853123 PMC4031057

[B21] Krizhevsky A. Sutskever I. Hinton G. E. (2017). Imagenet classification with deep convolutional neural networks. Commun. ACM. 60, 84–90. 10.1145/3065386

[B22] Krummen D. E. Baykaner T. Schricker A. A. Kowalewski C. A. Swarup V. Miller J. M.. (2017). Multicentre safety of adding Focal Impulse and Rotor Modulation (FIRM) to conventional ablation for atrial fibrillation. Europace 19, 769–774. 10.1093/europace/euw377 28339546 PMC5834113

[B23] Lawson C. L. (1984). C^1^ surface interpolation for scattered data on a sphere. Rocky Mountain J. Math. 14, 177–202. 10.1216/RMJ-1984-14-1-177

[B24] Li Z. Feng X. Wu Z. Yang C. Bai B. Yang Q. (2019). Classification of atrial fibrillation recurrence based on a convolution neural network with svm architecture. IEEE Access. 7, 77849–77856. 10.1109/ACCESS.2019.2920900

[B25] Lip G. Y. Tse H.-F. (2007). Management of atrial fibrillation. Lancet 370, 604–618. 10.1016/S0140-6736(07)61300-2 17707756

[B26] Luongo G. Azzolin L. Schuler S. Rivolta M. W. Almeida T. P. Martínez J. P.. (2021). Machine learning enables non-invasive prediction of atrial fibrillation driver location and acute pulmonary vein ablation success using the 12-lead ECG. Cardiovasc. Digit. Health J. 2, 126–136. 10.1016/j.cvdhj.2021.03.002 33899043 PMC8053175

[B27] MacLeod R. S. Brooks D. H. (1998). Recent progress in inverse problems in electrocardiology. Biol. Soc. Mag. 17, 73–83. 10.1109/51.646224 9460623

[B28] McHugh M. L. (2012). Interrater reliability: the kappa statistic. Biochem. Med. 22, 276–282. 10.11613/BM.2012.031 PMC3900052 23092060

[B29] Milanic M. Jazbinsek V. MacLeod R. S. Brooks D. H. Hren R. (2014). Assessment of regularization techniques for electrocardiographic imaging. J. Electrocardiol. 47, 20–28. 10.1016/j.jelectrocard.2013.10.004 24369741 PMC4154607

[B30] Narayan S. M. Krummen D. E. Shivkumar K. Clopton P. Rappel W.-J. Miller J. M. (2012). Treatment of atrial fibrillation by the ablation of localized sources. J. Am. Coll. Cardiol. 60, 628–636. 10.1016/j.jacc.2012.05.022 22818076 PMC3416917

[B31] Navara R. Leef G. Shenasa F. Kowalewski C. Rogers A. J. Meckler G.. (2018). Independent mapping methods reveal rotational activation near pulmonary veins where atrial fibrillation terminates before pulmonary vein isolation. J. Cardiovasc. Electrophysiol. 29, 687–695. 10.1111/jce.13446 29377478 PMC5980723

[B32] Nygren A. Fiset C. Firek L. Clark J. W. Lindblad D. S. Clark R. B.. (1998). Mathematical model of an adult human atrial cell: the role of k+ currents in repolarization. Circ. Res. 82, 63–81. 10.1161/01.RES.82.1.63 9440706

[B33] Oesterlein T. Frisch D. Loewe A. Seemann G. Schmitt C. Dössel O.. (2016). Basket-type catheters: diagnostic pitfalls caused by deformation and limited coverage. Biomed. Res. Int. 2016:5340574. 10.1155/2016/5340574 28070511 PMC5187596

[B34] Oster H. S. Rudy Y. (1992). The use of temporal information in the regularization of the inverse problem in electrocardiography. IEEE Trans. Biomed. Eng. 39:65–75. 10.1109/10.108129 1572683

[B35] Pan S. J. Yang Q. (2009). A survey on transfer learning. IEEE Trans. Knowl. Data Eng. 22, 1345–1359. 10.1109/TKDE.2009.191

[B36] Pedron-Torrecilla J. Climent A. Millet J. Berne P. Brugada J. Brugada R.. (2011). Characteristics of inverse-computed epicardial electrograms of brugada syndrome patients. Annu. Int. Conf. IEEE Eng. Med. Biol. Soc. 2011, 235–238. 10.1109/IEMBS.2011.6090044 22254293

[B37] Pedrón-Torrecilla J. Rodrigo M. Climent A. Liberos A. Pérez-David E. Bermejo J.. (2016). Noninvasive estimation of epicardial dominant high-frequency regions during atrial fibrillation. J. Cardiovasc. Electrophysiol. 27, 435–442. 10.1111/jce.12931 26776725 PMC5547887

[B38] Pourbabaee B. Roshtkhari M. J. Khorasani K. (2018). Deep convolutional neural networks and learning ECG features for screening paroxysmal atrial fibrillation patients. IEEE Trans. Syst. Man Cybernet. Syst. 48, 2095–2104. 10.1109/TSMC.2017.2705582

[B39] Rodrigo M. (2016). Non-invasive identification of atrial fibrillation drivers (Ph.D. thesis). Tesis doctoral). Universitat Politècnica de València, Valencia, Espa na.

[B40] Rodrigo M. Climent A. M. Liberos A. Calvo D. Fernández-Avilés F. Berenfeld O.. (2016). Identification of dominant excitation patterns and sources of atrial fibrillation by causality analysis. Ann. Biomed. Eng. 44, 2364–2376. 10.1007/s10439-015-1534-x 26850022 PMC5568434

[B41] Rodrigo M. Climent A. M. Liberos A. Fernández-Avilés F. Berenfeld O. Atienza F.. (2017). Technical considerations on phase mapping for identification of atrial reentrant activity in direct-And inverse-computed electrograms. Circ. Arrhythm. Electrophysiol. 10:e005008. 10.1161/CIRCEP.117.005008 28887361

[B42] Rodrigo M. Guillem M. S. Climent A. M. Pedrón-Torrecilla J. Liberos A. Millet J.. (2014). Body surface localization of left and right atrial high-frequency rotors in atrial fibrillation patients: a clinical-computational study. Heart Rhythm 11, 1584–1591. 10.1016/j.hrthm.2014.05.013 24846374 PMC4292884

[B43] Rottner L. Bellmann B. Lin T. Reissmann B. Tönnis T. Schleberger R.. (2020). Catheter ablation of atrial fibrillation: state of the art and future perspectives. Cardiol. Therapy 9, 45–58. 10.1007/s40119-019-00158-2 31898209 PMC7237603

[B44] Russakovsky O. Deng J. Su H. Krause J. Satheesh S. Ma S.. (2015). ImageNet large scale visual recognition challenge. Int. J. Comput. Vis. 115, 211–252. 10.1007/s11263-015-0816-y

[B45] Szegedy C. Ioffe S. Vanhoucke V. (2016). Inception-v4, inception-resnet and the impact of residual connections on learning. CoRR, abs/1602.07261.

[B46] Tikhonov A. N. Arsenin V. Y. (1977). Solutions of Ill-Posed Problems. New York, NY: Wiley.

[B47] Xia Y. Wulan N. Wang K. Zhang H. (2018). Detecting atrial fibrillation by deep convolutional neural networks. Comput. Biol. Med. 93, 84–92. 10.1016/j.compbiomed.2017.12.007 29291535

[B48] Xiong Z. Stiles M. K. Zhao J. (2017). Robust ecg signal classification for detection of atrial fibrillation using a novel neural network, in 2017 Computing in Cardiology (CinC) (Rennes: IEEE), 1–4.

